# Supplementary material for: Fast, In Vivo Model for Drug-Response Prediction in Patients with B-Cell Precursor Acute Lymphoblastic Leukemia
Source: Cancers (Basel). 2020 Jul 13;12(7):1883. doi: 10.3390/cancers12071883 (PMC7408814; doi:10.3390/cancers12071883)
Supplement: Supplementary file 1 [file cancers-12-01883-s001.pdf]

Supplementary Materials

Fast, In Vivo Model for Drug-Response Prediction in Patients with B-Cell Precursor Acute Lymphoblastic Leukemia

Anton Gauert, Nadine Olk, Helia Pimentel-Gutiérrez, Kathy Astrahantseff, Lasse D. Jensen, Yihai Cao, Angelika Eggert, Cornelia Eckert and Anja I.H. Hagemann

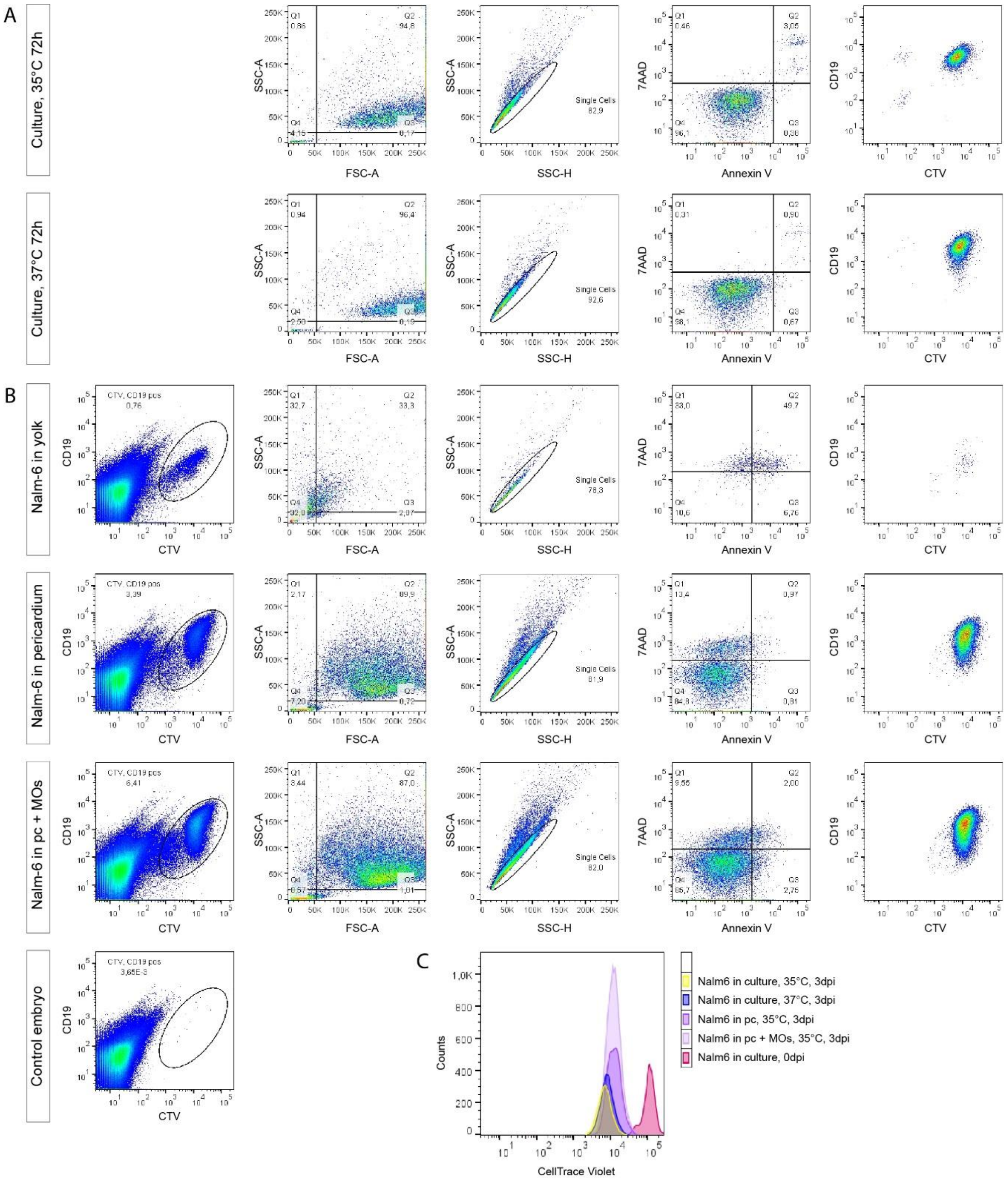

Figure S1. Gating strategy and flow cytometric analysis of Nalm-6 cells following 2D culture or engraftment in zebrafish embryos. A. Nalm-6 cells were prelabeled with CellTrace Violet (CTV) before culture. Nalm-6 cells were cultured on tissue culture plastic at the indicated temperatures for

72 h before analysis. Intact lymphoblasts were gated for single cells, and viable cells were selected from the Annexin/7AAD-negative fraction then evaluated for CD19 expression and CTV label intensity. B. Nalm-6 cells prelabeled with CTV and grown at 35°C for 72 h as grafts in host zebrafish embryos. Engraftment site indicated as well as whether the host embryo was transiently immunosuppressed using morpholinos (MOs). Groups of 10 embyos from each treatment group were pooled before single-cell dissociation for flow cytometric analysis. Gating strategy shown in A was applied, and viable graft cell fraction identified and quantified. To do this, graft cells were separated from auto-fluorescent zebrafish cells (only population present in control embryo shown in bottom row) to sort out the graft cell population for analysis. CTV labeling intensity and CD19 expression was analyzed in this graft cell population. Control embryos not engrafted show auto-fluorescence (lower panel). Pc = pericardium. Initial gating strategy for graft cell population is also shown in Figure 1 of the main text. C. Mean intensity of CTV labels from viable, CD19-positive cell selection only. Note the shift of CTV intensity after 3 days (3dpi).

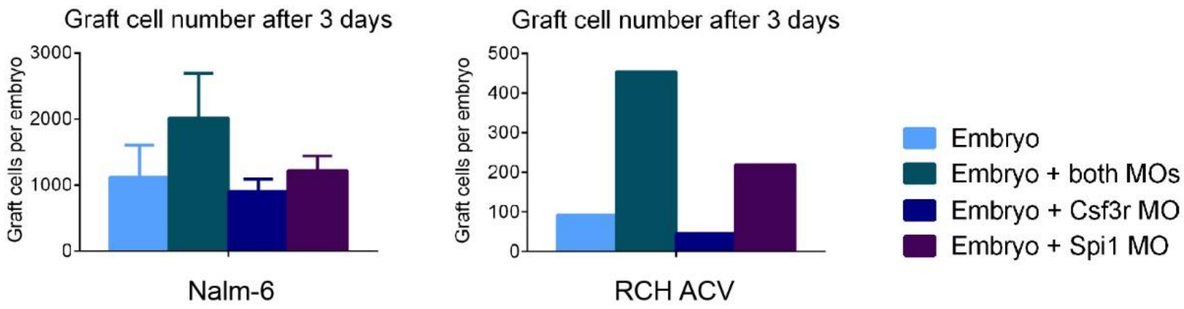

Figure S2. The impact of morpholino-induced transient host immunosuppression on Nalm-6 and RCH-ACV graft expansion. Mean graft cell numbers per embryo are shown after host immunosuppression via injection of morpholinos (MOs) against *spi1* and *csf3r* or only a single morpholino. Morpholinos were injected into fertilized eggs. Graft cell number per host embryo was calculated from flow cytometric analyses from groups of 10–20 pooled embryos from 2 independent experiments for Nalm-6 or 1 experiment for RCH-ACV grafts. Error bars indicate ± SEM.

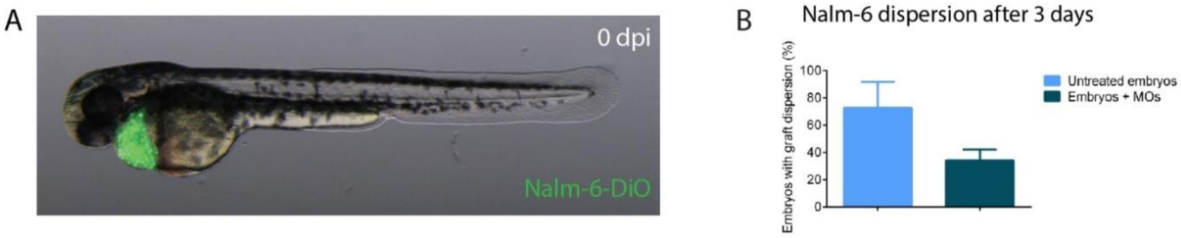

Figure S3. Intact host immune system promotes graft cell dissemination. **A**. Two-day old zebrafish embryo with green fluorescent DiO-labeled Nalm-6 graft in the pericardium shortly after injection. **B**. Mean percentage of embryos with DiO-labeled Nalm-6 graft cell dissemination at 3 days post-injection (dpi) with or without immunosuppression prior to injection counted from 3 independent experiments with 30 embryos each as depicted in Figure 1C. Error bars represent SEM, p-value = 0.074 in a paired t-test.

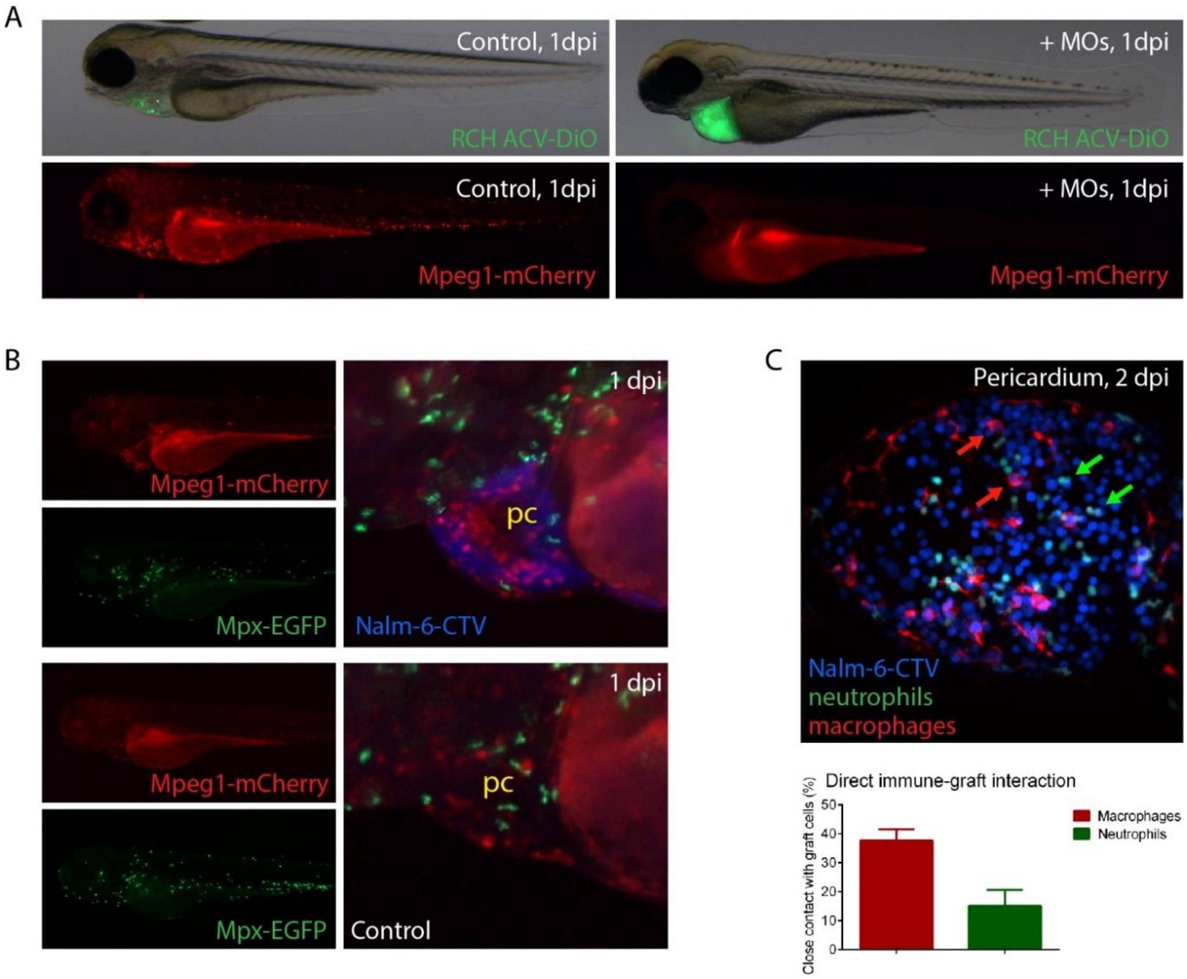

Figure S4. Host macrophages and neutrophils interact differently with ALL xenografts at the injection site. **A.** Morpholinos (MOs) against *spi1/csf3r* were injected into fertilized eggs of the tg(mpeg1-mCherry) reporter zebrafish line (red signal) to suppress macrophage development. Both embryos were transplanted with DiO labelled RCH-ACV cells (green signal) at 2 days post-fertilization. Green signal shows graft expansion at 3 days post-fertilization in the immunosuppressed fish (right) compared to the uninjected control (left). Single red cells are macrophages present in the control (left) but absent in the immunosuppressed embryo (right). **B**. Fluorescent images of double transgenic, live embryos with fluorescent macrophages (red, mpeg1-mCherry) and neutrophils (green, mpx-EGFP) represented as single channels or merge with engrafted CTV-labeled Nalm-6 cells (blue) into the pericardium (pc) or control without graft. Caudal is to the right and ventral to the bottom, all zebrafish embryos shown are not transiently immunosuppressed. **C**. Merged fluorescent high-resolution image of pericardium (ventral view) of a double-transgenic, live embryo as shown in B, engrafted with CTV-labeled Nalm-6 cells 2dpi. Arrows indicate macrophages (red) and neutrophils (green) in close contact with graft cells (blue). Bars indicate mean (n = 8) percentage of macrophages and neutrophils present at the graft site that interact closely with Nalm-6 cells at 2dpi. Error bars represent SEM.

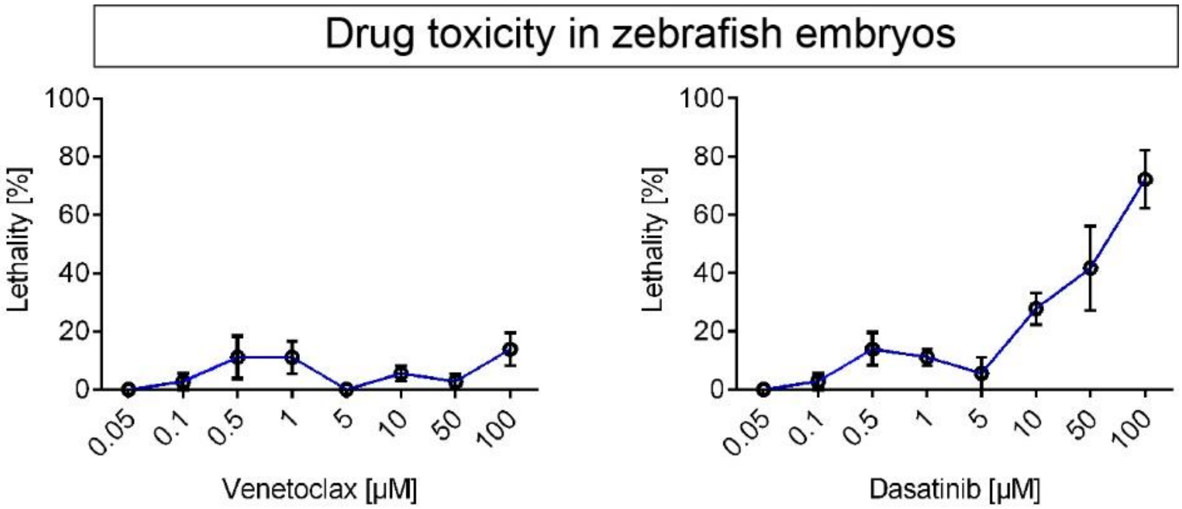

Figure S5. Drug toxicity in zebrafish embryos. Venetoclax was not toxic/lethal to zebrafish embryos until 100 µM was reached. LC50 for dasatinib on zebrafish embryos after 3 days at 35°C was assessed to be between 50 and 100µM in 2 - 3 independent experiments with at least 10 embryos each. Error bars represent SEM.

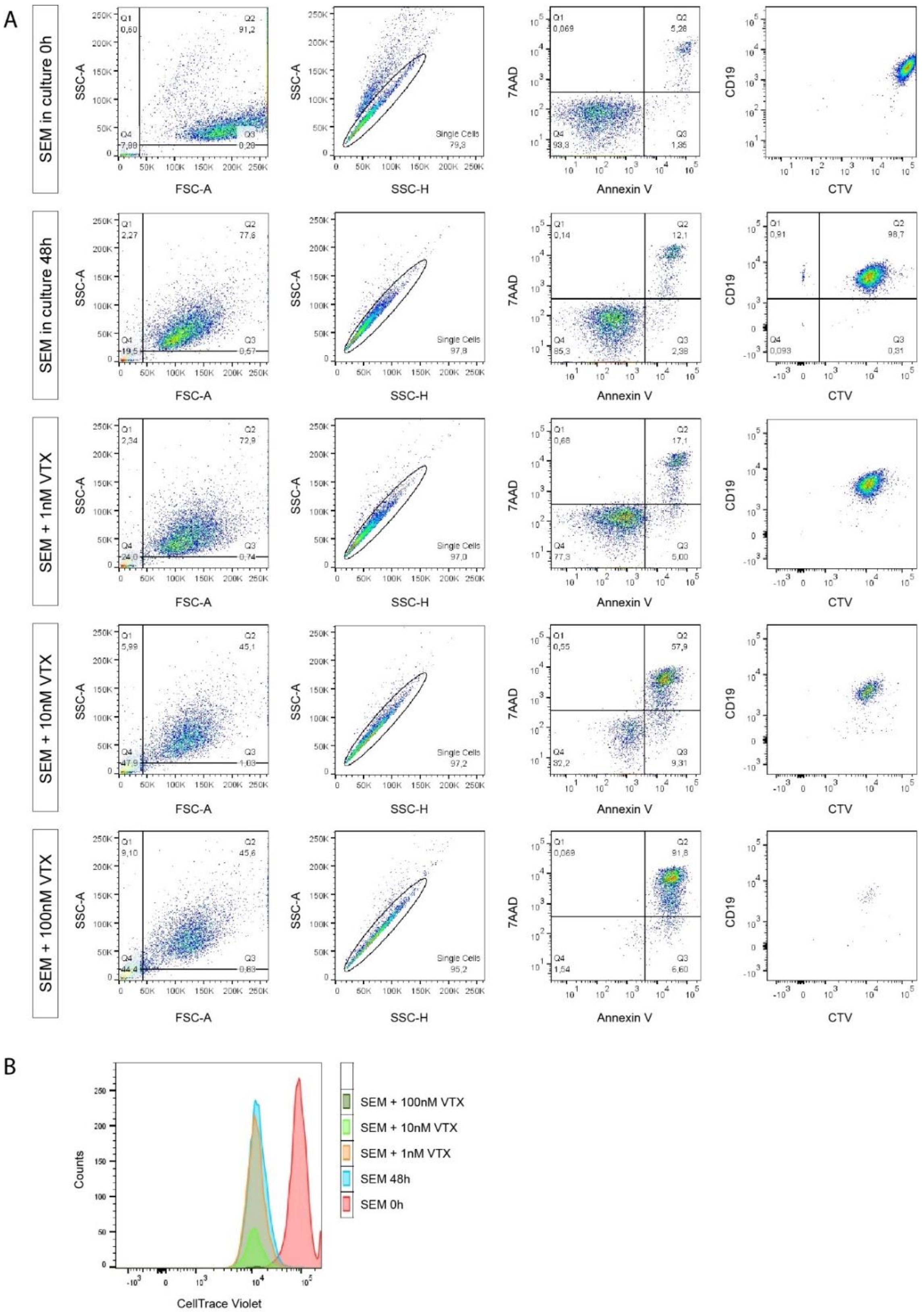

Figure S6. Gating strategy and flow cytometric analysis for SEM cells in 2D culture under different venetoclax treatment conditions. **A**. SEM cells were prelabeled with CellTrace Violet (CTV) before culture. Intact SEM cells were gated for single cells and viable cells were selected from the Annexin/7AAD-negative fraction. First 2 rows show dot plots for SEM cells after the indicated times in culture without venetoclax, and the 3 rows below show SEM cells treated with the indicated venetoclax dose after 48 h. A final gate for CD19-positive cells was added to assess the CD-19 marker in the population of viable cells. **B**. CellTrace Violet intensities are shown as peaks for CD19-positive

viable cell populations prior to treatment (0h) and after 48 h of treatment as indicated. Cell divisions were calculated from geometric mean intensities of CTV labels.

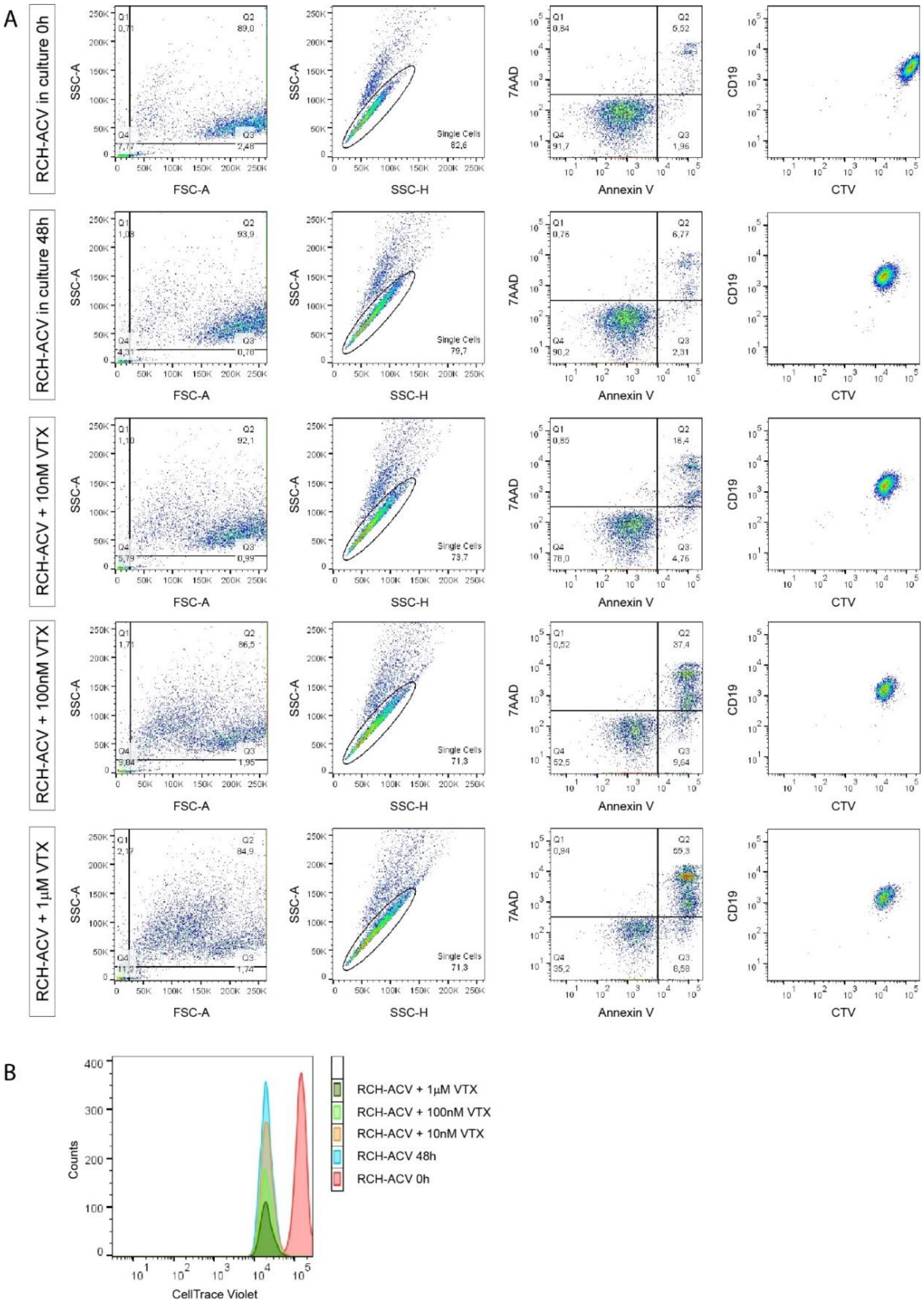

Figure S7. Gating strategy and flow cytometric analysis for RCH-ACV cells in 2D culture under different venetoclax treatment conditions. **A**. RCH-ACV cells were prelabeled with CellTrace Violet (CTV) before culture. Intact RCH-ACV cells were gated for single cells and viable cells were selected from the Annexin/7AAD-negative fraction. First 2 rows show dot plots for RCH-ACV cells after the indicated times in culture without venetoclax, and the 3 rows below show RCH-ACV cells treated

with the indicated venetoclax dose after 48 h. A final gate for CD19-positive cells was added to assess the CD19 marker in the population of viable cells. **B**. CellTrace Violet intensities are shown as peaks for CD19-positive viable cell populations prior to treatment (0 h) and after 48 h of treatment as indicated. Cell divisions were calculated from geometric mean intensities of CTV labels.

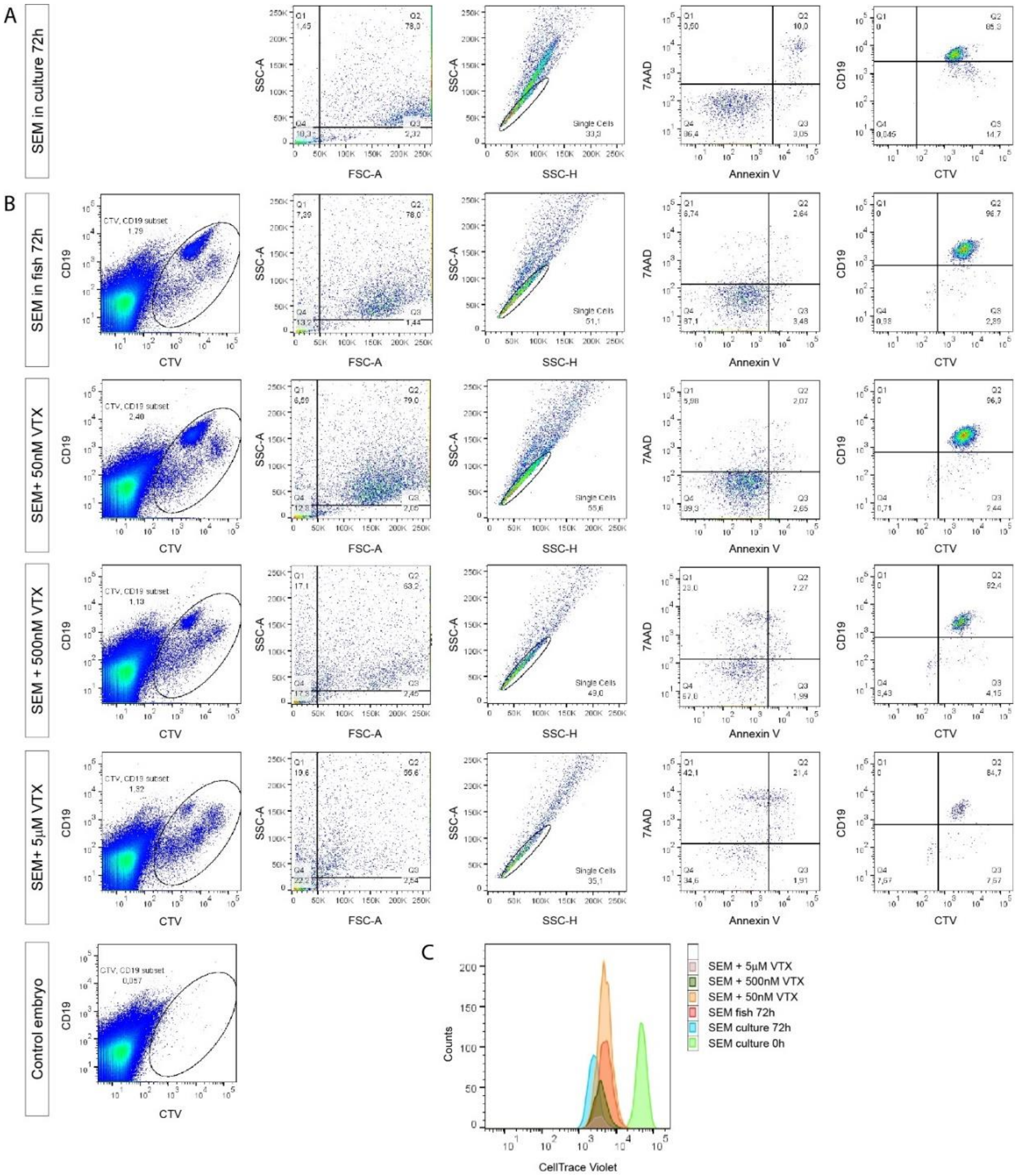

Figure S8. Gating strategy and flow cytometric analysis for dissociated zebrafish embryos from ALL-ZeFiX assays with engrafted SEM cells following 72-h testing with a range of venetoclax concentrations. **A.** Control SEM cells in 2D culture for 72h without venetoclax are shown. SEM cells were prelabeled with CellTrace Violet (CTV) before culture. Intact SEM cells were gated for single cells and viable cells were selected from the Annexin/7AAD-negative fraction. A final gate for CD19-positive cells was added to assess the CD19 marker in the population of viable cells. **B.** Dot plots illustrating gating strategies are shown for the total cell populations dissociated from ~10 pooled embryos treated in ALL-ZeFiX assays (engrafted with SEM cells) treated with the indicated venetoclax concentrations (untreated control, top row). Bottom row shows cells dissociated from a single zebrafish embryo of the same age that was not engrafted to illustrate the auto-fluorescent zebrafish cell population. All zebrafish embryos used in these experiments were transiently

immunosuppressed using the dual-morpholino (against *spi1* and *csf3r*) strategy. SEM cells were prelabeled with CTV prior to engraftment to track the viable graft cell population in the mixture of dissociated cells. Gating strategy first separated auto-fluorescent zebrafish cells from CTV-labeled graft cells. Only the graft cell population is then further analyzed with the same gates as in the parallel experiment with cells in conventional 2D culture; viable cells were selected from the Annexin/7AAD-negative fraction then evaluated for CD19 expression and CTV label intensity to track the number of cell divisions from the original engrafted population. **C.** CellTrace Violet intensities are shown as peaks for CD19-positive viable cell populations prior to treatment (0h) and after 72 h of treatment as indicated. Cell divisions were calculated from geometric mean intensities of CTV labels.

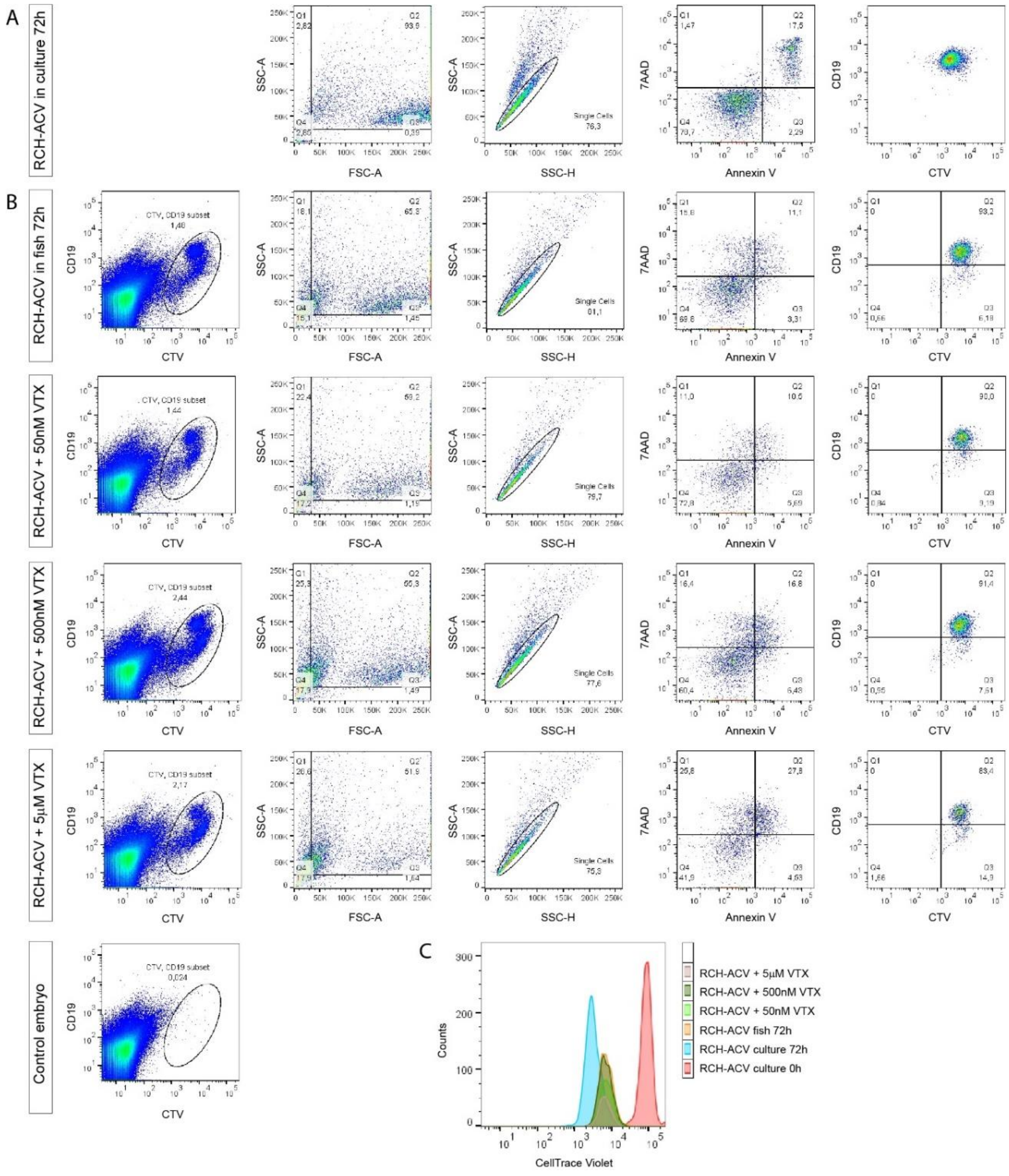

Figure S9. Gating strategy and flow cytometric analysis for dissociated zebrafish embryos from ALL-ZeFiX assays with engrafted RCH-ACV cells following 72-h testing with a range of venetoclax concentrations. A. Control RCH-ACV cells in 2D culture for 72h without venetoclax are shown. RCH-ACV cells were prelabeled with CellTrace Violet (CTV) before culture. Intact RCH-ACV cells were gated for single cells and viable cells were selected from the Annexin/7AAD-negative fraction. A final gate for CD19-positive cells was added to assess the CD19 marker in the population of viable cells. B.

Dot plots illustrating gating strategies are shown for the total cell populations dissociated from ~10 pooled embryos treated in ALL-ZeFiX assays (engrafted with RCH-ACV cells) treated with the indicated venetoclax concentrations (untreated control, top row). Bottom row shows cells dissociated from a single zebrafish embryo of the same age that was not engrafted to illustrate the auto-fluorescent zebrafish cell population. All zebrafish embryos used in these experiments were transiently immunosuppressed using the dual-morpholino (against *spi1* and *csf3r*) strategy. RCH-ACV cells were prelabeled with CTV prior to engraftment to track the viable graft cell population in the mixture of dissociated cells. Gating strategy first separated auto-fluorescent zebrafish cells from CTV-labeled graft cells. Only the graft cell population is then further analyzed with the same gates as in the parallel experiment with cells in conventional 2D culture; viable cells were selected from the Annexin/7AAD-negative fraction then evaluated for CD19 expression and CTV label intensity to track the number of cell divisions from the original engrafted population. C. CellTrace Violet intensities are shown as peaks for CD19-positive viable cell populations prior to treatment (0h) and after 72 h of treatment as indicated. Cell divisions were calculated from geometric mean intensities of CTV labels.

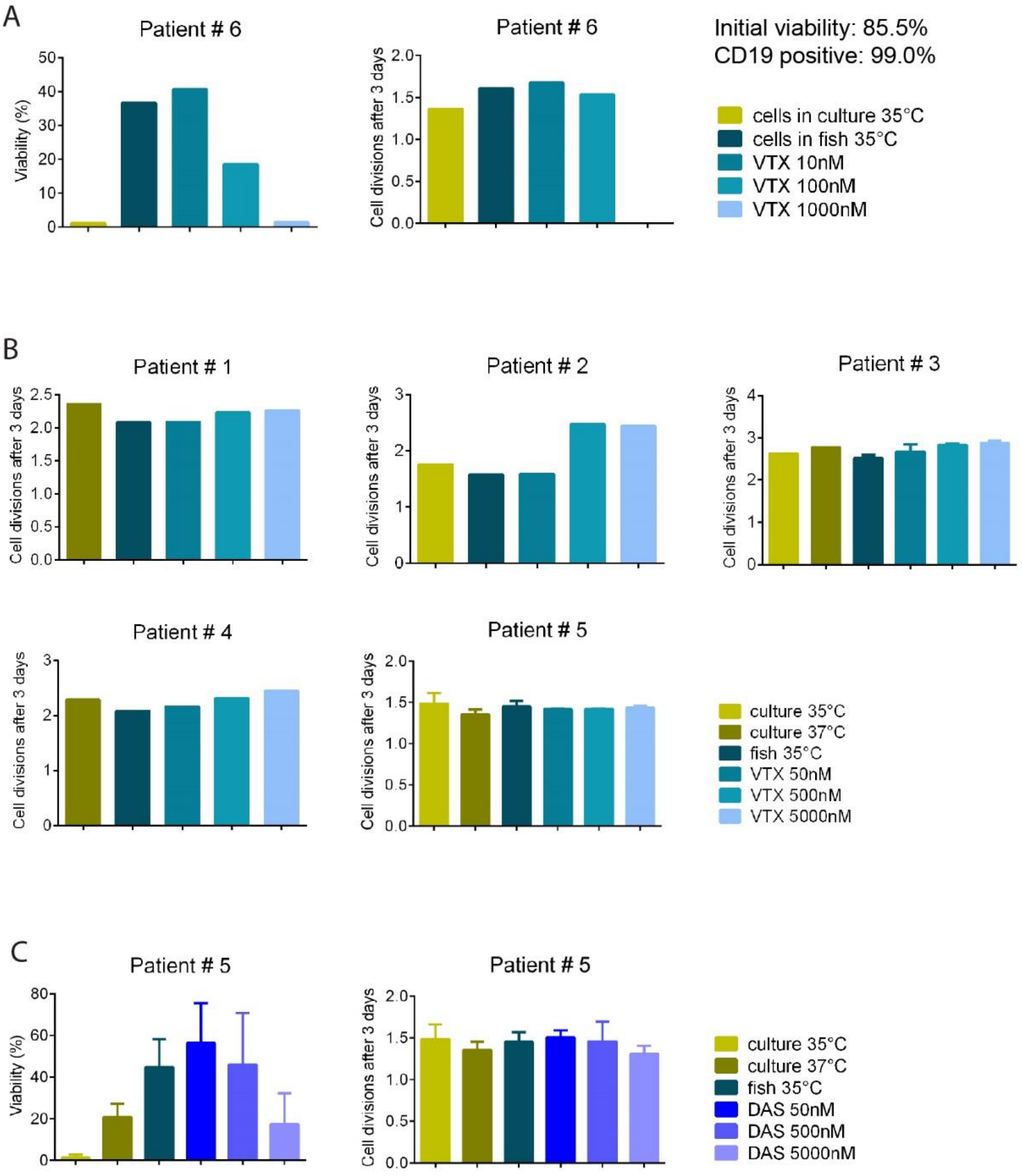

Figure S10. Proliferation rate of patient-derived engrafted cells in the ALLZeFix assay is comparable to in vitro 2D cultures after 3 days. **A**. Graft cell (from patient 6) viability and cell divisions were flow

cytometrically assessed after 3 days of treatment with venetoclax. Residual bone marrow sample provided only enough blast cells for 1 experiment pooled from 10–20 engrafted zebrafish embryos. **B**. Cell divisions completed by the BCP-ALL graft after 3 days of venetoclax treatment. Experimental results correspond to viability measurements shown in Figure 3. **C**. BCP-ALL cells from patient 5 were tested for their sensitivity to dasatinib (DAS) treatment. Viability and cell divisions were flow cytometrically assessed after 3 days of dasatinib treatment. Data from 2 independent experiments. Error bars represent SEM.

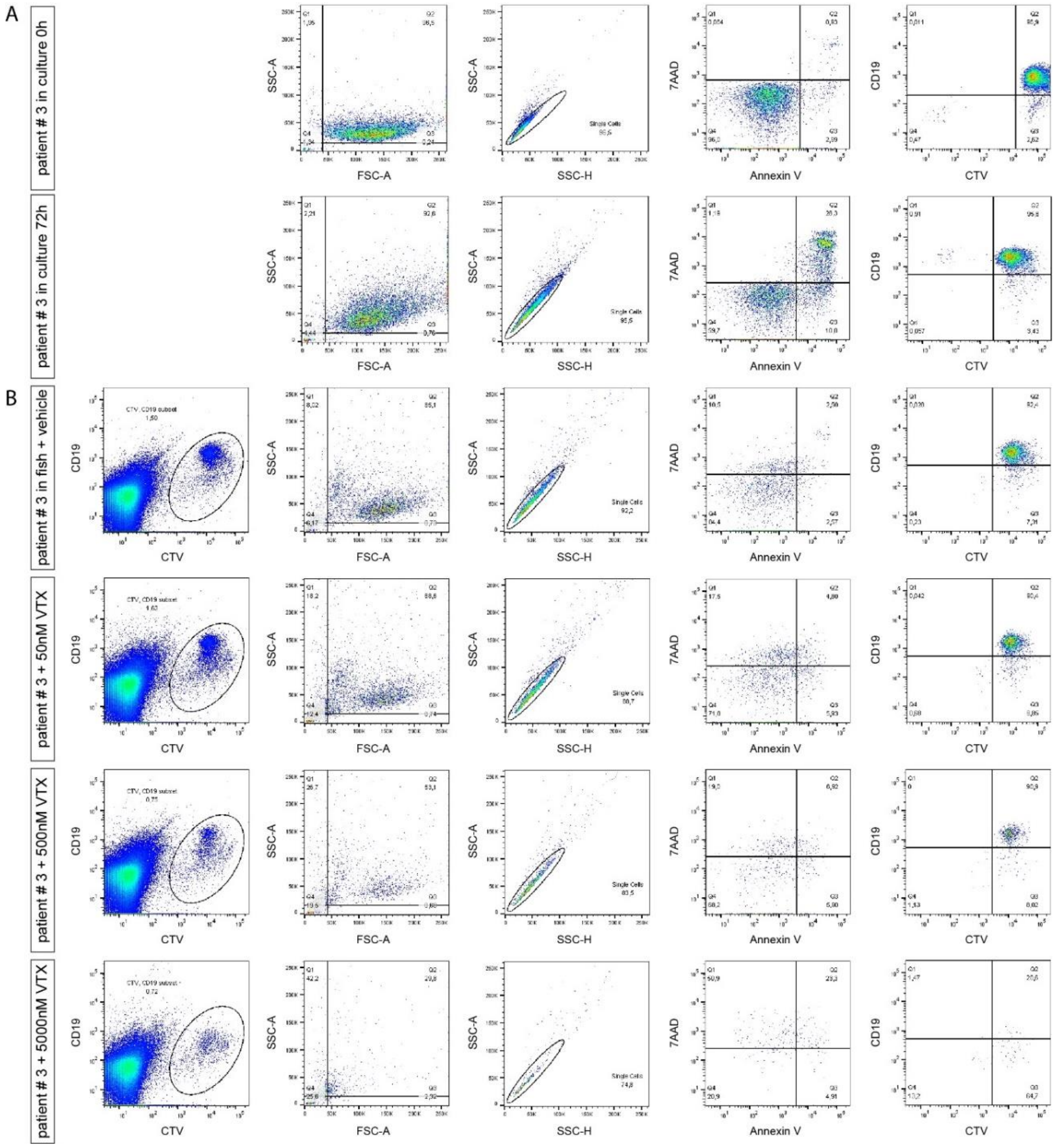

Figure S11. Gating strategy and flow cytometric analysis of BCP-ALL cells from patient 3 following 2D culture or ALL-ZeFix assay with a range of venetoclax concentrations. **A**. Primary blast cells from a bone marrow sample collected from patient 3 at the time point documented in Table S1 were prelabeled with CellTrace Violet (CTV) and cultured on tissue-culture plastic for the times indicated. Intact cells were gated for single cells, and viable cells were selected from the Annexin/7AAD-negative fraction. A final gate for CD19-positive cells was added to determine the proportion of blast cells retaining CD19 expression. **B**. Dot plots are shown for each step of the gating strategy of total cell populations dissociated from ~10 pooled zebrafish embryos following ALL-ZeFix assay completion (treatment indicated left of each row). All zebrafish embryos used in these experiments

were transiently immunosuppressed using the dual-morpholino (against *spi1* and *csf3r*) strategy. Primary blast cells cells were prelabeled with CTV prior to engraftment to track the viable graft cell population in the mixture of dissociated cells. Gating strategy first separated auto-fluorescent zebrafish cells from CTV-labeled graft cells. Intact lymphoblasts were gated for single cells, and viable cells were selected from the Annexin/7AAD-negative fraction then evaluated for CD19 expression and CTV label intensity. Cell divisions were calculated from geometric mean intensities of CTV labels.

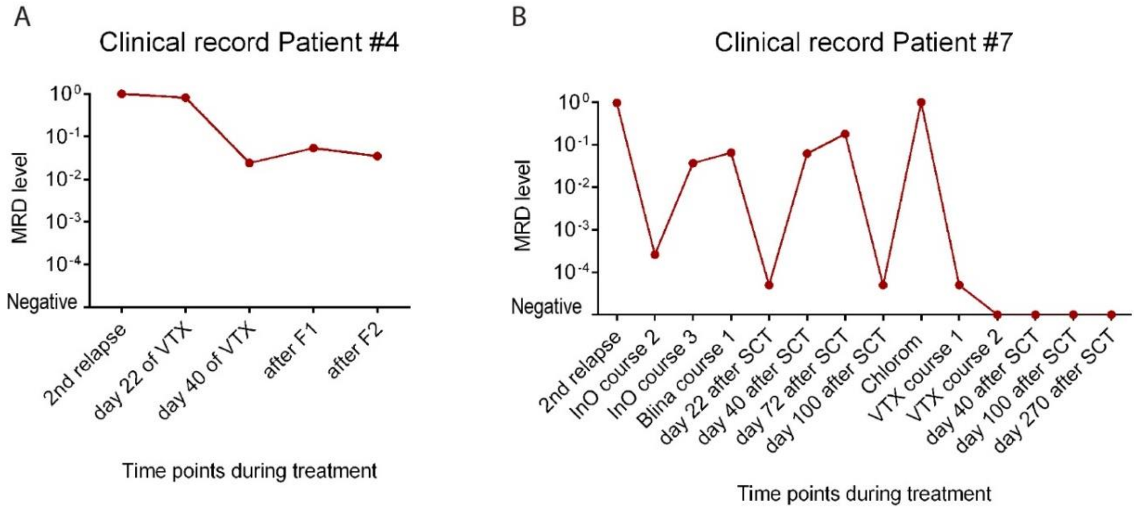

Figure S12. Poor and good venetoclax responses in two patients with refractory chemotherapy-resistant BCP-ALL. **A**. Minimal residual disease (MRD) is shown for patient 4 during and after venetoclax treatment. The persisting MRD (>10^{-2}) following both the first venetoclax therapy block and second therapy block with venetoclax combined with vincristine and dexamethasone is classified as a poor response. Two induction courses, according to the ALL-REZ BFM 2002 trial protocol (F1, F2), were administered after each venetoclax course. Venetoclax was administered according to Place et al., 2018 [5]. Patient 4 died 3 months after completion of venetoclax treatment followed by 2 additional cycles of polychemotherapy and a course of immunotherapy. **B.** MRD is shown for patient 7 before, during and after venetoclax treatment. This patient did not respond to standard polychemotherapy during frontline and first relapse treatment. Therefore, immunotherapy (inotuzumab, InO and blinatumomab, Blina) was administered before stem cell transplantation. However, leukemia always rapidly reappeared. Day 150 after stem cell transplantation, the patient developed a chloroma in the occipital skull (left) and vitreous body (right), which responded completely to two courses of venetoclax (administered according to the protocol described in Place et al., 2018) in combination with chemotherapy (cyclophosphamide, 6-thioguanine und cytarabine), followed by a second stem cell transplantation. No MRD was detected for >1 year. Patient 7 is in continuous complete remission, and has been since 1 year, when a second stem cell transplantation was carried out after two courses of venetoclax in combination with chemotherapy (cyclophosphamide, 6-thioguanine und cytarabine).

Table S1. Clinical and genetic characteristics of patient samples used for engraftment into zebrafish.

**No.**	**Disease status at time of sample**	**Immuno-phenotype**	**Time point of relapse**	**Site of relapse**	**Genetics**	**Response to induction treatment**	**ALL-ZeFiX graft success**
**1**	1st relapse	BCP-ALL	very early	Bone marrow/ CNS	B-other	Good, MRD <10^{-3}	yes
**2**	Primary	Biphenotypic leukemia	NA	NA	*KMT2A/AFF1*	Poor response	yes
**3**	1st relapse	BCP-ALL	very early	Bone marrow/ CNS	*KMT2a/AFF1, TP53 mut.*	Poor, MRD ≥10^{-2}	yes
**4**	2nd relapse	BCP-ALL	22 months after 1st relapse	Bone marrow	B-other	Good, MRD <10^{-3} at 1st relapse	yes
**5**	1st relapse	BCP-ALL	early	Bone marrow/ CNS	*BCR/ABL1*	Good, MRD <10^{-3}	yes
**6**	1st relapse	BCP-ALL	very early	Bone marrow	iAMP21	Nonresponder	yes
**7**	2nd relapse	BCP-ALL	6 months after 1st relapse	Bone marrow	*TCF3/PBX1*, TP53 mut.	Nonresponder	yes
**8**	1st relapse	BCP-ALL	late	Bone marrow/testis	*ETV6/RUNX1*	Good, MRD <10^{-3}	yes
**9**	1st relapse	BCP-ALL	late	Bone marrow	B-other	Good, MRD <10^{-3}	yes
**10**	1st relapse	BCP-ALL	early	Bone marrow/ CNS/testis	B-other	Good, MRD <10^{-3}	no
**11**	1st relapse	BCP-ALL	late	Bone marrow	B-other, *IgH/CRLF2*	Poor, MRD ≥10^{-2}	no
**12**	1st relapse	BCP-ALL	late	Bone marrow	unknown	Good, MRD <10^{-3}	no
**13**	1st relapse	BCP-ALL	late	Bone marrow	*BCR/ABL1*	Unknown	no
**14**	1st relapse	BCP-ALL	late	Bone marrow/ CNS	*BCR/ABL1*	Poor, MRD ≥10^{-2}	no
**15**	1st relapse	BCP-ALL	very early	Bone marrow	unknown	Poor, MRD ≥10^{-2}	no

BCP-ALL, B-cell precursor acute lymphoblastic leukemia; CNS, central nervous system; MRD, minimal residual disease; NA, not applicable. Definitions for relapse time points: very early, < 18 months after initial diagnosis; early, ≥18 months after initial diagnosis and <6 months after cessation of frontline treatment; late, ≥ 6 months after cessation of frontline treatment; nonresponse, ≥5% leukemic blast cells after induction treatment and the following two treatment courses. Disease status at time of sample indicates when sample collection occurred; always prior to chemotherapy.

© 2020 by the authors. Licensee MDPI, Basel, Switzerland. This article is an open access article distributed under the terms and conditions of the Creative Commons Attribution (CC BY) license (http://creativecommons.org/licenses/by/4.0/).
